# miRNA Expression Profiles and Potential as Biomarkers in Nontuberculous Mycobacterial Pulmonary Disease

**DOI:** 10.1038/s41598-020-60132-0

**Published:** 2020-02-21

**Authors:** Sun Ae Han, Byung Woo Jhun, Su-Young Kim, Seong Mi Moon, Bumhee Yang, O. Jung Kwon, Charles L. Daley, Sung Jae Shin, Won-Jung Koh

**Affiliations:** 1 Division of Pulmonary and Critical Care Medicine, Department of Medicine, Samsung Medical Center, Sungkyunkwan University School of Medicine, Seoul, South Korea; 2 Division of Mycobacterial and Respiratory Infections, Department of Medicine, National Jewish Health, Denver, CO USA; 3 Department of Medicine, University of Colorado, Aurora, Colorado USA; 4 Department of Microbiology, Institute for Immunology and Immunological Disease, Brain Korea 21 PLUS Project for Medical Science, Yonsei University College of Medicine, Seoul, South Korea

**Keywords:** miRNAs, Bacterial infection

## Abstract

Pulmonary disease (PD) due to nontuberculous mycobacteria (NTM) is increasing globally, but specific biomarkers for NTM-PD have not been established. As circulating miRNAs are promising biomarkers for various diseases, we investigated whether miRNAs have potential as NTM-PD biomarkers. Sera from 12 NTM-PD patients due to *Mycobacterium avium*, *M. intracellulare*, *M. abscessus*, or *M. massiliense* and three healthy controls were initially evaluated via small RNA sequencing. Multiple miRNAs showed significant differences in expression in patients compared to in healthy controls, with some expression differences unique to PD caused by a specific mycobacterial species. Notably, 14 miRNAs exhibited significant expression differences in PD associated with all four mycobacteria. Validation by quantitative reverse-transcription-PCR in an additional 40 patients with NTM-PD and 40 healthy controls confirmed that four differentially expressed miRNAs (hsa-miR-484, hsa-miR-584-5p, hsa-miR-625-3p, and hsa-miR-4732-5p) showed significantly higher serum expressions in NTM-PD patients than in controls. Receiver operating characteristic curve analysis of these four miRNAs supported the discriminative potential for NTM-PD and their combination provided an improved diagnostic value for NTM-PD. Furthermore, bioinformatics analysis revealed their 125 target genes, which were mostly associated with immune responses. Collectively, this study identified four miRNAs as potential biomarkers for NTM-PD and provided insight into NTM-PD pathophysiology.

## Introduction

Nontuberculous mycobacteria (NTM) are mycobacteria other than the *Mycobacterium tuberculosis* complex and *Mycobacterium leprae*. More than 180 officially recognized NTM species have been identified, with the most frequent human pathogens associated with pulmonary disease (PD) due to NTM belonging to the *Mycobacterium avium* complex (MAC), followed by *M. abscessus* (MAB)^1,2^. MAC primarily comprises *M. avium* and *M. intracellulare*, and MAB primarily comprises *M. abscessus* subspecies *abscessus* (hereafter referred to as *M. abscessus*) and *M. abscessus* subspecies *massiliense* (hereafter referred to as *M. massiliense*)^1,2^. Despite the global increase in the burden of NTM-PD in recent decades, there is limited information regarding specific biomarkers for distinguishing patients with NTM-PD from healthy individuals or studies of the disease pathophysiology^3,4^.

MicroRNAs (miRNAs) are small, 18–25-nucleotide, endogenous, stable, highly conserved noncoding RNAs with important functions in post-transcriptional gene regulation under both physiological and pathological conditions^5,6^. Numerous studies have reported the aberrant expression of several miRNAs in various conditions, including cancer and infectious diseases. miRNAs reportedly play an important regulatory role in the pathogenesis or progression of infectious diseases^7,8^ and serve as potential modulators of innate and adaptive immune responses^9,10^. Serum miRNAs are not readily degraded by enzymes, unaffected by changes in temperature and time, and resistant to acids and alkalis^11,12^. Furthermore, serum miRNAs are stable, and evaluating serum miRNA profiles is a feasible diagnostic procedure in clinical laboratories. Because of their high diagnostic potential, serum miRNAs have been evaluated as biomarkers in several pathological conditions including TB, with some studies achieving 82% to 100% accuracy in diagnosing TB by evaluating miRNA^13–18^. Additionally, several studies have reported differences in miRNA levels among patients with active TB, latent TB, and healthy controls^18,19^. Recently, the roles of miRNAs in mycobacterial infections, particularly in tuberculosis (TB), has received increasing attention^13,20–23^ Such studies have reported that specific miRNAs are differentially expressed in macrophages or patients infected with *M. tuberculosis* complex and that several of these miRNAs interact with their cognate target genes. Additionally, studies have demonstrated that miRNAs are involved in the progression of TB and that serum miRNA profiles can be useful as potential diagnostic or therapeutic biomarkers in patients with TB^13,20–23^. The serum levels of miR-144 and miR-155, which were differentially expressed in patients with pulmonary TB compared to in healthy controls, have been reported to be diagnostic markers for TB^24,25^.

As the burden of NTM-PD is increasing worldwide, studies reporting on the expression of miRNA in NTM-PD have also increased. For example, overexpression of let-7e, miR-29a, and miR-886-5p inhibited *M. avium*-induced apoptosis in human monocyte-derived macrophages by modulating caspases 3 and 7^26^. Serum miR-346, expressed in *M. avium*-infected macrophages secreted into the bloodstream, was quantitatively controlled by bacterial load and identified as a potential biomarker of MAC-PD^27^. Although circulating miRNAs were recently recognized as promising disease biomarkers in infectious diseases, few studies have examined the role of miRNA in NTM-PD. Therefore, we evaluated serum miRNAs that are differentially expressed in NTM-PD patients compared to in healthy controls by RNA sequencing and explored the potency of serum miRNA expression profiles as discriminating markers for NTM-PD.

## Results

### Patients

In total, 95 participants were recruited in this study, including 52 patients with NTM-PD and 43 healthy volunteers (Table 1). The median age of the patients was 56 years, and 45 (87%) were female. The median body mass index of the 52 patients was 20.8 kg/m^2^, and 46 (89%) were never smokers. Among the patients with NTM-PD, the most common underlying respiratory disease was bronchiectasis (n = 48, 92%), followed by previous treated TB (n = 17, 33%). The median value of white blood cell count and erythrocyte sedimentation rate was 6040/µL and 36 mm/hr, respectively. Out of the 52 patients with NTM-PD, 48 (92%) had nodular bronchiectatic form, and 4 (8%) had the fibrocavitary form (Supplementary Fig. S1). The median forced expiratory volume in 1 second (%) on pulmonary function test at diagnosis was 79%. In the 43 healthy controls, the median age was 48 years, and 29 (67%) patients were female.

**Table 1:** Demographic and clinical characteristics of study participants.

Characteristics	NTM-PD (n = 52)	Healthy Controls (n = 43)
Age (years)	56 (50–62)	48 (40–56)
Female sex	45 (87)	29 (67)
Body mass index (kg/m^2^)	20.8 (19.0–22.5)	NA
Never smoker	46 (89)	INA
**Etiologic organism**		INA
*M. avium*	13 (25)	—
*M. intracellulare*	14 (27)	—
*M. abscessus*	13 (25)	—
*M. massiliense*	12 (23)	—
**Underlying disease**		
Bronchiectasis	48 (92)	—
Previously treated tuberculosis	17 (33)	—
Previous thoracic surgery	1 (2)	—
Chronic heart disease	1 (2)	—
Chronic liver disease	1 (2)	—
**Laboratory data**		INA
White blood cell (/µL)	6040 (4780–7813)	INA
Lymphocyte (%)	29.9 (19.5–34.6)	INA
Erythrocyte sedimentation rate (mm/hr)	36 (18–52)	INA
Albumin (g/dl)	4.5 (4.3–4.6)	INA
Computed tomography finding		—
Nodular bronchiectatic form	48 (92)	—
Fibrocavitary form	4 (8)	—
Positive acid fast stain	31 (60)	INA
Forced expiratory volume in 1 second (%)	79 (72–93)	INA

Data are presented as no. (%) or median (interquartile range). NTM-PD, nontuberculous mycobacterial pulmonary disease; INA, information not available.

### Illumina-based small RNA sequencing of serum miRNAs

Serum levels of miRNA from 12 patients with NTM-PD, including patients infected with *M. avium*, *M. intracellulare*, *M. abscessus*, or *M. massiliense* (n = 3 each), and three healthy individuals were examined by Illumina small RNA sequencing in the discovery phase. Averages of 11,217,363 and 18,556,427 reads of RNAs ranging from 18 to 30 nucleotides were obtained from pooled serum samples of patients with NTM-PD and healthy controls, respectively. The length distribution of clean sequences in the reference genome was determined. Length distribution analysis of serum pools from both patients with NTM-PD and healthy controls revealed that most reads were in the range of 18–24 nt, which is consistent with the common length of miRNAs^28,29^. The three pools of serum samples contained small RNAs of various lengths (Supplementary Fig. S2). Thereafter, bioinformatics analysis was performed to investigate the small RNA species and sequencing frequencies.

In serum derived from patients with NTM-PD and healthy controls, multiple and heterogeneous small RNA species, including miRNAs, long intergenic noncoding RNA, ribosomal, small nucleolar, and small nuclear RNA were identified (Table 2). In serum samples of patients with NTM-PD and healthy controls, miRNAs accounted for 39.7% and 44.5%, respectively, of the total amount of small RNAs (sequencing reads), and there was no significant difference between the two groups. We analyzed all clean reads using mirDeep software to identify known and novel miRNAs. In total, 467 and 407 known miRNAs were identified among patients with NTM-PD and healthy controls, respectively (Table 2). Novel miRNA precursors were predicted, and an average of 262 miRNAs in patients with NTM-PD and 193 miRNAs in healthy controls were identified (Table 2).

**Table 2:** The number of miRNAs in serum samples from patients with NTM-PD and healthy controls by small RNA sequencing analysis.

	NTM-PD	Healthy controls
Total reads	11,217,363	18,556,427
Precursor miRNA reads	3,445	8,444
Mature miRNA reads	7,250,677	5,265,841
No. of known miRNAs	467	407
No. of novel miRNAs	262	193
**Distribution of the genome-mapped sequence reads**		
miRNA	3,517,419 (39.7)	3,083,932 (44.5)
lincRNA	2,397,431 (27.0)	1,655,179 (23.9)
rRNA	10,857 (0.1)	6,326 (0.1)
snoRNA	8,987 (0.1)	5,626 (0.1)
snRNA	25,265 (0.3)	20,821 (0.3)
others	2,924,390 (32.8)	2,164,554 (31.2)
**Distribution of genomic repetitive sequences**		
LINE (sense)	280,607 (27.0)	237,082 (26.4)
LINE (anti sense)	155,079 (14.9)	136,196 (15.2)
LTR (sense)	148,237 (14.3)	134,467 (15.0)
LTR (anti sense)	105,699 (10.2)	108,196 (12.1)
SINE (sense)	124,916 (12.0)	101,246 (11.3)
SINE (anti sense)	118,899 (11.4)	87,079 (9.7)
Retroposon (sense)	39675 (3.8)	39720 (4.4)
Retroposon (anti sense)	64934 (6.3)	52578 (5.9)
others	766 (0.1)	696 (0.1)

Data are presented as no. (%). NTM-PD, nontuberculous mycobacterial pulmonary disease; miRNA, microRNA; lincRNA, long intergenic noncoding RNA; rRNA, ribosomal RNA; snoRNA, small nucleolar RNA; snRNA, small nuclear RNA; LINE, long interspersed nuclear element; LTR, long terminal repeat; SINE short interspersed nuclear element.

**Figure 1:**
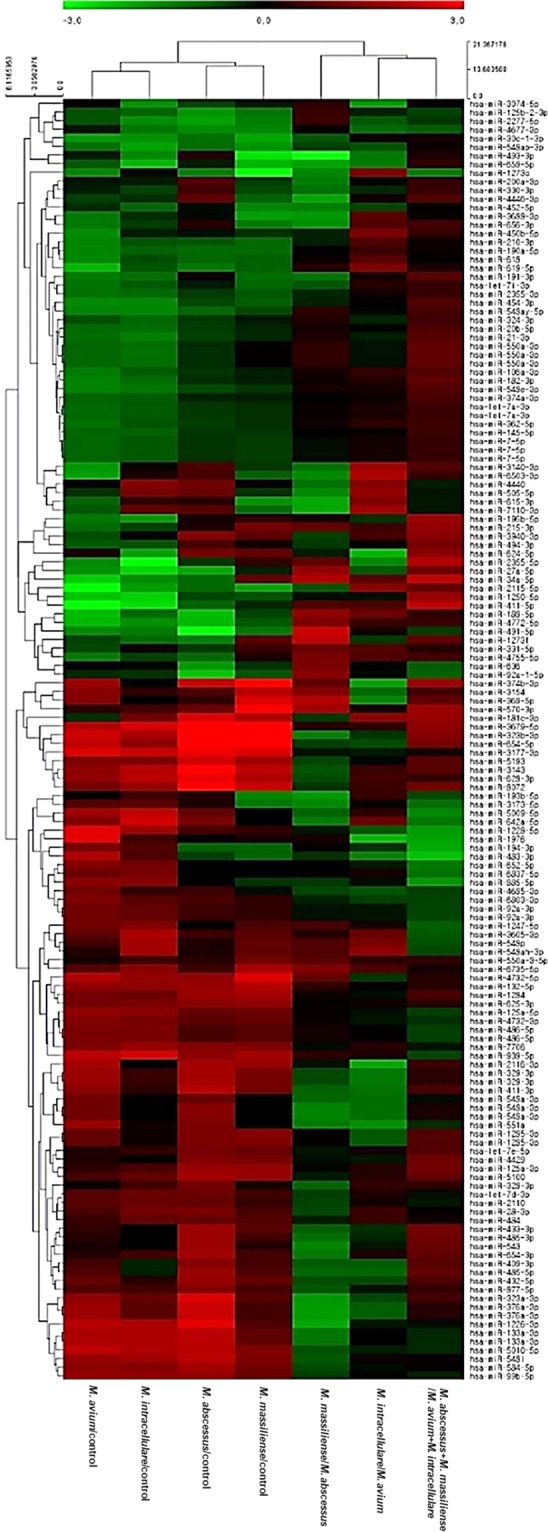
Hierarchical clustering of differentially expressed microRNAs in sera of patients with NTM-PD and healthy controls. A total of 148 miRNAs were differentially expressed with separate clustering of NTM-PD patients and healthy controls. Each row represents the expression of a given gene in each sample, and each column represents the expression of each gene in a given sample. Rows and columns are hierarchically clustered. Red and Green colors represent up- and downregulation of miRNAs in NTM-PD patients relative to levels in healthy controls.

**Figure 2:**
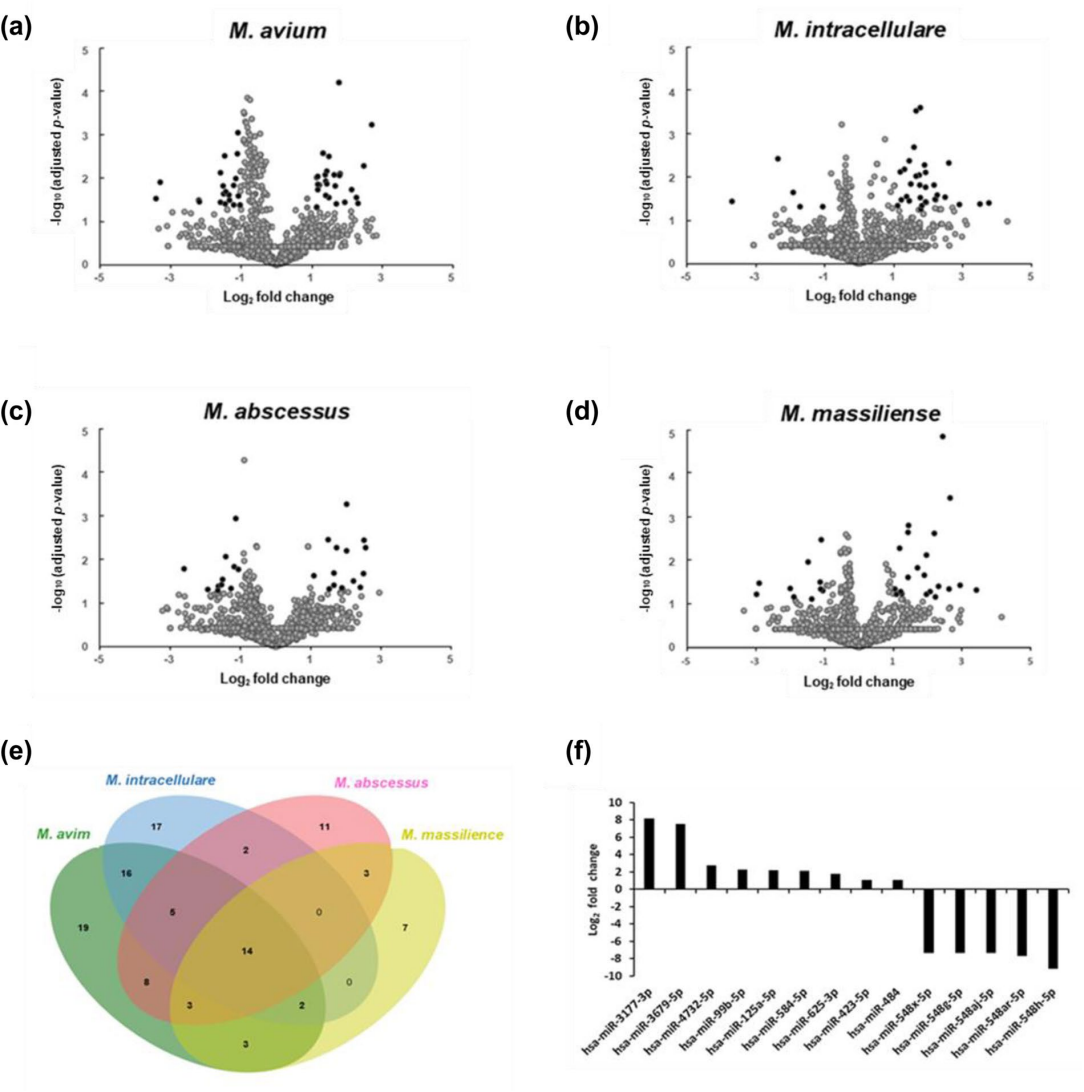
Volcano plots and Venn diagram of differentially expressed miRNAs in NTM-PD. (**a**–**d**) Volcano plots representing log2 fold change as a function of –log_10_ (the adjusted p-value) for miRNA expression in NTM-PD versus healthy controls. miRNAs found to be differentially expressed between the NTM-PD patients and controls are shown in black and unchanged miRNAs are shown in grey. (**a**) *Mycobacterium avium* patients, (**b**) *M. intracellulare* patients, (**c**) *M. abscessus* patients, and (**d**) *M. massiliense* patients. (**e**) Venn diagram showing miRNAs significantly expressed in NTM-PD patients versus healthy controls. (**f**) Histogram of the 14 miRNAs that were differentially expressed in NTM-PD from all four NTM species. Expression is shown as fold change over levels in healthy controls.

In addition, differentially expressed miRNAs were observed in the serum miRNA profiles of patients with NTM-PD relative to in healthy controls (Fig. 1). We identified 148 differentially expressed miRNAs based on two-fold changes and p-values less than 0.05. Of these 148 miRNAs, 70 miRNAs showed significant differences in expression in patients with *M. avium*-PD, 56 miRNAs in patients with *M. intracellulare*-PD, 46 miRNAs in patients with *M. abscessus*-PD, and 32 miRNAs in patients with *M. massiliense*-PD compared to in healthy controls (Supplementary Tables S1–4, Fig. 2a–d). However, only 14 of these miRNAs were differentially expressed in all four patient groups, i.e., in PD due to any of the four NTM organisms (*M. avium*, *M. intracellulare*, *M. abscessus*, and *M. massiliense*) (Fig. 2e,f).

### Differential expression of miRNAs via qRT-PCR analysis

**Figure 3:**
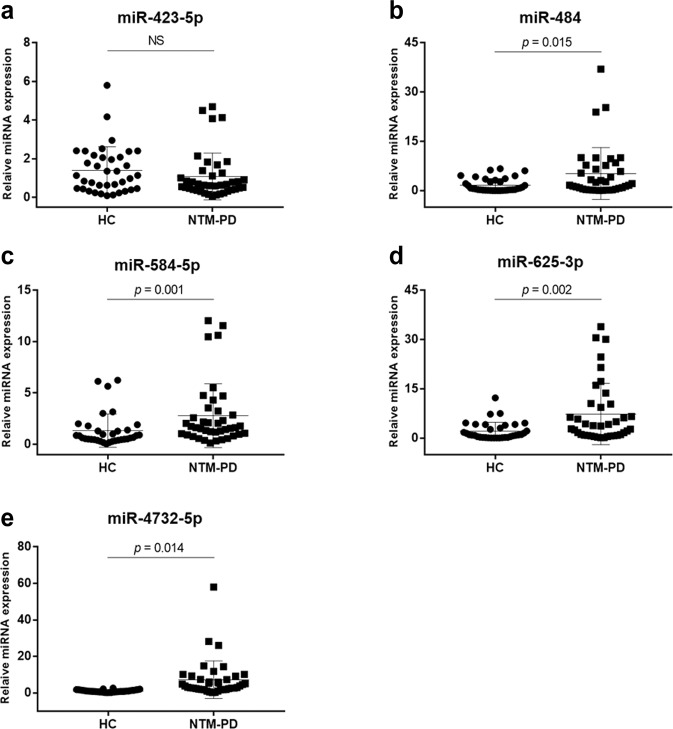
Validation of selected miRNA expression levels by qRT-PCR in NTM-PD patients. Serum levels of the five miRNAs were measured in 40 NTM-PD patients and in 40 healthy controls. Expression levels of the miRNAs are normalized to miR-16. (**a**) has-miR-423-5p, (**b**) has-miR-484, (**c**) has-miR-584-5p, (**d**) has-miR-625-3p, and (**e**) has-miR-4732-5p. qRT-PCR, quantitative real-time PCR; HC, healthy controls; NTM-PD, nontuberculous mycobacterial pulmonary disease; NS, not significant.

As the 14 miRNAs with differential expression in all four patient groups were considered as potential biomarkers, their expression was verified by qRT-PCR analysis. In the selection phase, the 14 candidate miRNAs were measured in a separate set of individual serum samples from 12 patients with NTM-PD and three healthy controls, i.e., the number of the same subjects used for small RNA sequencing. miRNAs with a mean fold-change (NTM-PD/healthy controls) of >2 and *p*-values < 0.05 were selected for further analysis. The analysis indicated that five miRNAs (hsa-miR-423-5p, hsa-miR-484, hsa-miR-584-5p, hsa-miR-625-3p, and hsa-miR-4732-5p) of the 14 candidates showed significant differences in expression between patients with NTM-PD and healthy controls (Fig. 3).

qRT-PCR-based validation was performed to quantify differences in the expression levels of the five miRNAs in samples from 40 healthy controls and 40 patients with NTM-PD (including NTM-PD due to *M. avium*, *M. intracellulare*, *M. abscessus*, and *M. massiliense*; n = 10 each). Apart from hsa-miR-423-5p (*p* = 0.116), four of the five miRNAs, i.e., hsa-miR-484 (*p* = 0.015), hsa-miR-584-5p (*p* = 0.001), hsa-miR-625-3p (*p* = 0.002), and has-miR-4732-5p (*p* = 0.014), showed significantly higher serum expression levels in patients with NTM-PD than in healthy controls (Fig. 3).

### Receiver operating characteristic (ROC) curve analysis of four miRNAs

**Figure 4:**
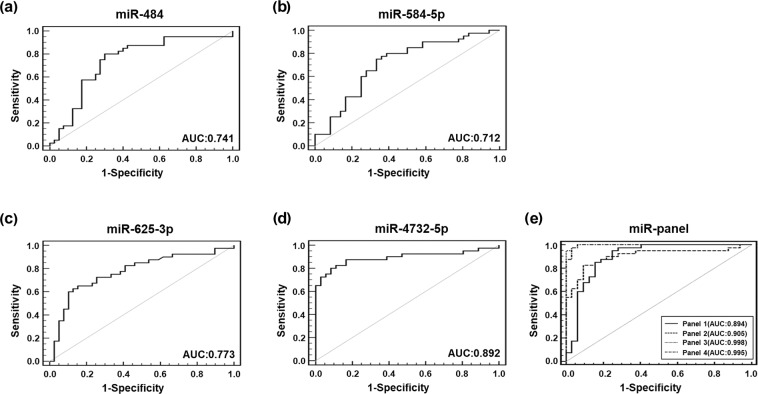
Receiver operating characteristic (ROC) curves of four selected miRNAs in NTM-PD patients. The ROC curves were used to show the discriminative ability of the selected four miRNAs with sensitivity (true-positive rate) and 1-specificity (false-positive rate). The x-axis shows 1-specificity, and the y-axis shows sensitivity. Multivariate logistic regression analysis was performed, and ROC curves were generated to evaluate the ability of the chosen miRNAs to distinguish NTM-PD patients from controls. (**a**) has-miR-484, (**b**) has-miR-584-5p, (**c**) has-miR-625-3p, (**d**) has-miR-4732-5p, and (**e**) has-miR-panel. AUC, area under the curve; Panel 1, has-miR-484+ has-miR-584-5p+ has-miR-625-3p+ has-miR-4732-5p; Panel 2, has-miR-484+ has-miR-584-5p+ has-miR-4732-5p; Panel 3, has-miR-484+ has-miR-625-3p+ has-miR-4732-5p; Panel 4, has-miR-584-5p+ has-miR-625-3p+ has-miR-4732-5p.

Using validation data obtained from the 40 patients with NTM-PD and 40 healthy controls, ROC curve analysis was performed to determine how well the selected serum miRNAs could distinguish between the two groups. miR-423-5p (*p* = 0.116) were removed based on *p*-values > 0.05. ROC curves were constructed to compare the relative expression levels of the four miRNAs between the two groups (Table 3, Fig. 4). The areas under the curves (AUC) of has-miR-4732-5p for distinguishing NTM-PD from healthy controls was 0.892(*p* < 0.0001, 95% CI = 0.800–0.952), which was comparable to that of hsa-miR-484, 0.741 (*p* < 0.0001, 95% CI = 0.631–0.833), hsa-miR-584-5p, 0.712 (*p* < 0.0001, 95% CI = 0.597–0.810), and hsa-miR-625-3p, 0.773 (*p* < 0.0001, 95% CI = 0.665–0.860) (Fig. 4a–d). Multivariate logistic regression analysis in ROC curve was used to identify the optimal combination of four miRNAs to discriminate NTM-PD. The integration of has-miR-4732-5p with hsa-miR-484 and hsa-miR-625-3p (AUC 0.998; sensitivity 95%; and specificity 100%) resulted in an improved diagnostic value for NTM-PD (Fig. 4e).

**Table 3:** Comparison of the AUC, the sensitivity, and specificity for predicting NTM-PD.

miRNA	AUC (95% CI)	Sensitivity,%	Specificity,%
has-miR-484	0.741 (0.631–0.833)	80.0	70.0
has-miR-584-5p	0.712 (0.597–0.810)	75.0	66.7
has-miR-625-3p	0.773 (0.665–0.860)	60.0	89.7
has-miR-4732-5p	0.892 (0.800–0.952)	80.0	91.7
has-miR-4732-5p+ has-miR-484+ has-miR-584-5p+ has-miR-625-3p	0.894 (0.802–0.953)	95.0	71.4
has-miR-4732-5p+ has-miR-484+ has-miR-584-5p	0.905 (0.813–0.962)	82.5	90.6
has-miR-4732-5p+ has-miR-484+ has-miR-625-3p	0.998 (0.949–1.000)	95.0	100.0
has-miR-4732-5p+ has-miR-584-5p+ has-miR-625-3p	0.995 (0.941–1.000)	97.5	96.9
has-miR-4732-5p+ has-miR-484	0.908 (0.819–0.962)	87.5	86.1
has-miR-4732-5p+ has-miR-584-5p	0.888 (0.791–0.950)	80.0	90.6
has-miR-4732-5p+ has-miR-625-3p	0.996 (0.945–1.000)	97.5	97.2
has-miR-484+ has-miR-584-5p	0.776 (0.666–0.863)	80.0	69.4
has-miR-484+ has-miR-625-3p	0.810 (0.706–0.890)	65.0	87.2
has-miR-584-5p+ has-miR-625-3p	0.867 (0.769–0.935)	92.5	74.3

AUC, area under the curve; NTM-PD, nontuberculous mycobacterial pulmonary disease; miRNA, microRNA.

### Construction of miRNA-gene networks

**Figure 5:**
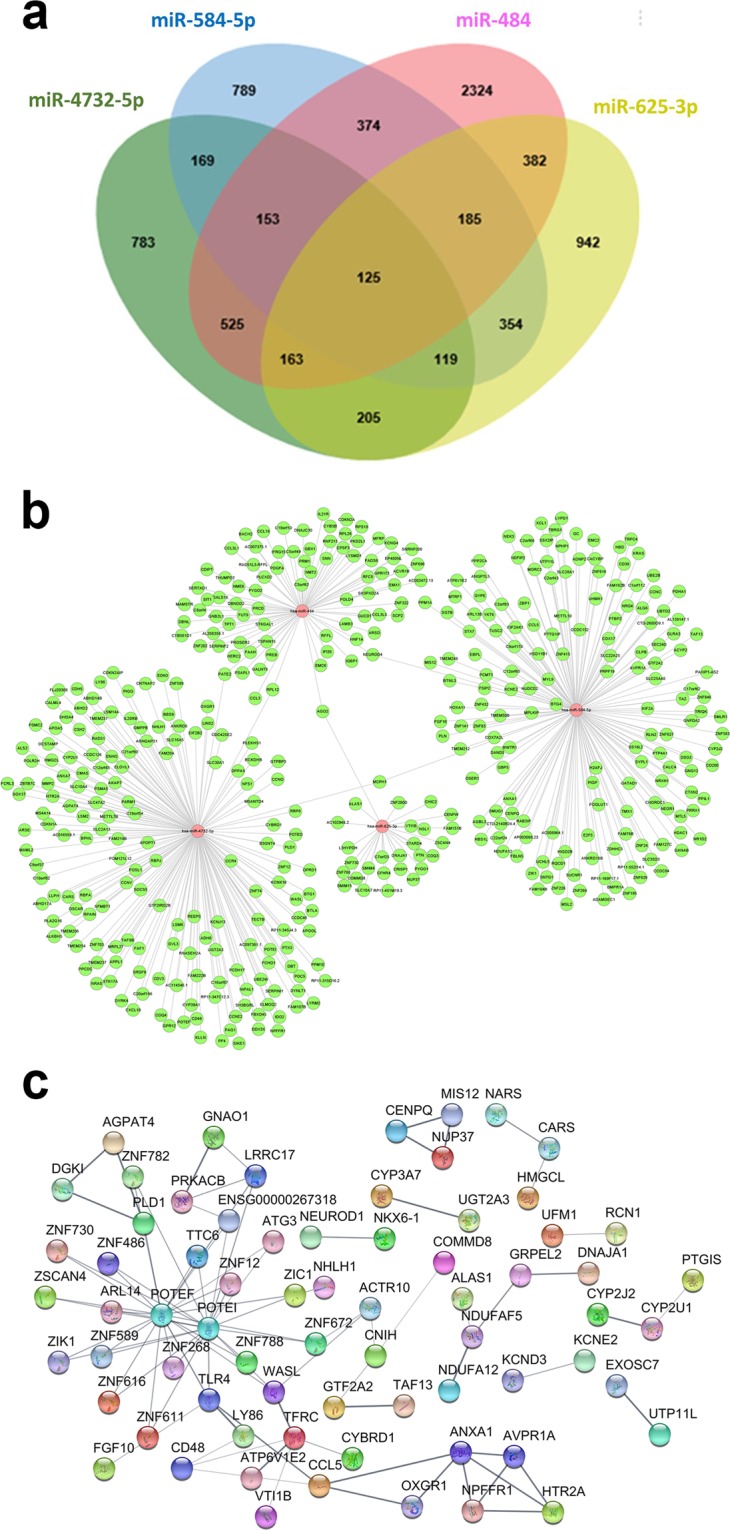
The miRNA-gene network for miRNAs associated with genes related to NTM-PD. (**a**) Venn diagram showing overlap of gene targets of hsa-miR-484, hsa-miR-584-5p, hsa-miR-625-3p, and hsa-miR-4732-5p. 125 genes were screened to be targeted by the four miRNAs. (**b**) miRNA-gene network for hsa-miR-484, hsa-miR-584-5p, hsa-miR-625-3p, and hsa-miR-4732-5p. The network was built using 125 targeted genes and predicted interactions from the TargetScan databases. Red represents miRNAs, and green represents genes; their relationship is represented by the edges. (**c**) Protein-protein interaction analysis performed using the Search Tool for the Retrieval of Interacting Genes database revealed that the target genes of hsa-miR-484, hsa-miR-584-5p, hsa-miR-625-3p, and hsa-miR-4732-5p may interact with each other. The disconnected nodules are hidden. Bold lines indicate stronger association.

To further investigate the putative functions of the candidate miRNAs, the TargetScan database (http://targetscan.org) was used to analyze the target mRNAs, and the Database for Annotation, Visualization, and Integrated Discovery (http://david.abcc.ncifcrf.gov/) was used to identify the biochemical pathways associated with the conserved targets. Figure 5 shows the miRNA-gene network for hsa-miR-484, hsa-miR-584-5p, hsa-miR-625-3p, and hsa-miR-4732-5p. These four miRNAs were associated with 4231, 2268, 2475, and 2242 target mRNAs in the TargetScan database, respectively. Among these genes, 125 genes, including nine genes related to TB, were predicted to be targeted by the four miRNAs in common (Fig. 5a–c). For example, hsa-miR-484, hsa-miR-584-5p, hsa-miR-625-3p, and hsa-miR-4732-5p all targeted the nuclear factor of activated T cells 5 (NFAT5) and Toll-like receptor 4 (TLR4). Hsa-miR-484, hsa-miR-584-5p, and hsa-miR-4732-5p targeted interleukin 6 (IL-6), and hsa-miR-484 and hsa-miR-625-3p targeted interleukin-17 (IL-17). Has-miR-484 targeted CD40 and the CD40 ligand. Hsa-miR-625-3p targeted CD28, a coreceptor that mediates T cell activation.

### Go analysis and KEGG pathway analysis of target genes

**Figure 6:**
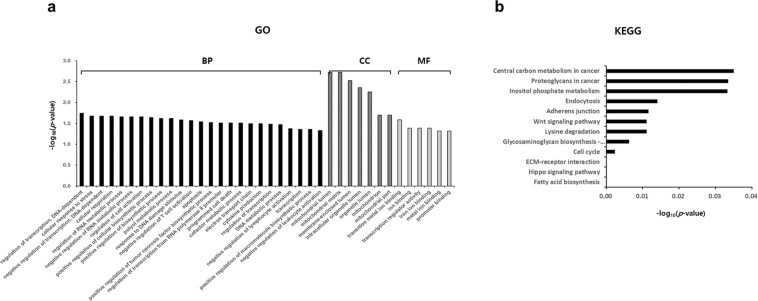
GO enrichment and KEGG pathway analysis for the target genes of selected miRNAs in NTM-PD. (**a**) GO analysis of hsa-miR-484, hsa-miR-584-5p, hsa-miR-625-3p, and hsa-miR-4732-5p target genes; the x-axis indicates -log_10_(p-value), and the y-axis indicates GO terms; GO, gene ontology; BP, biological processes (black); CC, cellular component (dark gray); MF, molecular function (light gray). (**b**) KEGG pathway analysis of hsa-miR-484, hsa-miR-584-5p, hsa-miR-625-3p, and hsa-miR-4732-5p target genes; the x-axis indicates -log_10_(p-value), and the y-axis indicates KEGG pathway categories.

Gene ontology (GO) and the Kyoto Encyclopedia of Genes and Genomes (KEGG) pathway analysis were applied to further classify the identified genes into the functional groups. GO enrichment analyses were performed to classify 125 target genes based on biological processes, molecular function, and cellular component (Fig. 6a). In the case of biological processes, the genes were classified into forty-six categories, of which the most significant term was regulation of transcription and DNA-template (GO: 0006355). Based on cellular components, the genes were classified into ten categories, of which the most significant term was mitochondrial lumen (GO: 0005759). Based on molecular function, the genes were classified into eleven categories, of which the most significant term was transition metal ion binding (GO: 0046914).

KEGG pathway analysis indicated that 125 putative target genes were mainly involved in central carbon metabolism in cancer, proteoglycans in cancer, inositol phosphate metabolism, endocytosis, adherent junction, Wnt signaling pathway, lysine degradation, glycosaminoglycan biosynthesis, cell cycle, ECM receptor interaction, hippo signaling pathway, and fatty acid biosynthesis (Fig. 6b).

## Discussion

This study was conducted to compare the expression profiles of circulating serum miRNA from NTM-PD and healthy controls. In the discovery phase, 70 miRNAs in patients with *M. avium*-PD, 56 miRNAs in patients with *M. intracellulare*-PD, 46 miRNAs in patients with *M. abscessus*-PD, and 32 miRNAs in patients with *M. massiliense*-PD showed significant differences in expression relative to levels in healthy controls. Differential expression of 14 of these miRNAs was common to PD caused by any of the four organisms. In the validation phase, using qRT-PCR analysis of sera from additional patients and controls, we confirmed that four miRNAs (hsa-miR-484, hsa-miR-584-5p, hsa-miR-625-3p, and hsa-miR-4732-5p) of the 14 miRNAs were differentially expressed in patients. We further determined that 125 gene targets were shared by the candidate miRNAs and were associated with NTM-PD on TargetScan analysis. The present study is the first to demonstrate that hsa-miR-484, hsa-miR-584-5p, hsa-miR-625-3p, and hsa-miR-4732-5p are associated with NTM-PD and are potential biomarkers for NTM-PD. This study is valuable because multiple patients with NTM-PD and disease due to several NTM organisms were examined.

In the present study, four serum miRNAs (hsa-miR-484, hsa-miR-584-5p, hsa-miR-625-3p, and hsa-miR-4732-5p) showed increased expression in patients with NTM-PD compared to in healthy controls. Interestingly, these four miRNAs were previously implicated in infection with *Mycobacterium bovis* bacillus Calmette-Guérin (BCG) and immune diseases. miR-484 is upregulated in exosomes of BCG-infected macrophages and targets mitochondrial cleavage protein 1 to modulate the intermediate metabolic pathway^30^. Various assays have revealed that miR-484 upregulation clearly influences cell migration and cellular proliferative capacity, and miR-484 is also tumorigenic, promoting the progression of non-small cell lung cancer^31^. miR-584 is reportedly upregulated in breast cancer cells, enhancing nuclear factor kappa B signaling, and inhibiting TGF-β signaling^32^. TGF-β has several inhibitory effects on T cells, B cells, macrophages, and other cells, and increased TGF-β levels are associated with protection and/or recovery from autoimmune diseases^33^. Previous studies reported that miRNA-625-3p in the blood facilitated the detection of malignancy in benign lung tumors^34^, and miRNA-625-3p is reportedly upregulated in the urine of pulmonary TB patients and is an excellent diagnostic urinary biomarker^35^. miR-4732-3p is associated with breast cancer and stomach cancer and is specifically expressed in exosomes released from macrophages after infection with BCG^20,36^. We hypothesized that differential expression of these miRNAs in patients with NTM-PD and healthy controls would result in differences of target mRNA expression; thus, additional experimental validation is further required.

In the present study, 125 genes were predicted to be targeted by the four miRNAs, including nine genes related to *M. tuberculosis* infection. Our results revealed that various genes associated with the innate immune response, cell proliferation, and apoptosis are potential targets of hsa-miR-484, hsa-miR-584-5p, hsa-miR-625-3p, and hsa-miR-4732-5p. Our results further showed that NFAT5 is a target gene for all four miRNAs; NFAT5 plays an important role in modulating HIV-1 replication by *M. tuberculosis* during co-infection, by directly interacting with the viral promoter^37^. Hsa-miR-484, hsa-miR-584-5p, and hsa-miR-4732-5p also targeted IL-6, a pleiotropic cytokine that plays a central role in host defense^38^. These results suggest that the four miRNAs play an important role in host-pathogen interactions.

We performed comprehensive GO enrichment and KEGG pathway analysis of the target genes of the differentially expressed miRNAs. GO analysis indicated that these genes are involved in immune responses and oxidation-reduction processes and in the regulation of tumor necrosis factor-α. KEGG analysis revealed the involvement of these genes in cell growth, migration, and proliferation and in pathways such as Hippo signaling, Wnt signaling, p53 signaling, and TGF-β signaling. Thus, these miRNAs may influence NTM infection, as they affect immune cell development. In addition, most proteins encoded by the target genes are involved in mitogen-activated protein kinase signaling, Wnt signaling, and TGF-β signaling in NTM-PD and may be useful in elucidating the pathogenesis of NTM-PD.

To further evaluate the diagnostic value of hsa-miR-484, hsa-miR-584-5p, hsa-miR-625-3p, and hsa-miR-4732-5p, we performed a more detailed analysis using ROC curves. The present study found that the ROC curve of the combined four differentially expressed miRNAs showed an optimal discriminative ability with an AUC of 0.998 (95% CI = 0.949–1.000), and diagnostic sensitivity and specificity of 95 and 100%, respectively. Our data showed that the diagnostic value of these four miRNAs combination was higher than that of the individual miRNAs, indicating that the combinational signature of these four miRNA could serve as discriminating markers for NTM-PD.

There are several limitations to the current study. First, the number of samples in this study was small. Second, the present study did not include patient subjects with other lung diseases as controls for NTM-PD. To further validate the four miRNAs in discriminating NTM-PD from other lung diseases, appropriate PD control groups are required to confirm whether our findings are unique to NTM-PD. Finally, the combination sets of miRNAs target prediction were solely based on TargetScan database. Thus, the use of efficient genetic algorithms for variable selection in logistic regression model, rather than a linear model, may have advantages to select the clinically optimal markers among all extracted data sets, more accurately^39^.

In conclusion, to our best knowledge, this is the first study to determine the serum miRNA profiles in NTM-PD through deep sequencing. We identified 14 differentially expressed miRNAs in patients with NTM-PD compared to in healthy individuals, including nine upregulated miRNAs and five downregulated miRNAs. The present study verified the relative expression levels of hsa-miR-484, hsa-miR-584-5p, hsa-miR-625-3p, and hsa-miR-4732-5p, indicating their potential as biomarkers for NTM-PD. GO enrichment and KEGG analysis showed that the target genes of the four differentially expressed miRNAs were mainly involved in the immune response. These results indicate the presence of a serum-specific miRNA signature in patients with NTM-PD, which may modulate host antibacterial pathways in response to NTM infection and can be considered a potential molecular tool for diagnosing NTM-PD.

## Materials and Methods

### Study population

Patients with newly diagnosed NTM-PD and who had sera stored at the time of diagnosis of NTM-PD were screened from the NTM Registry of Samsung Medical Center, a 1,979-bed referral hospital in Seoul, South Korea (ClinicalTrials.gov identifier: NCT00970801). To identify NTM-PD-specific miRNAs, we excluded patients with diseases that may influence miRNA expression such as chronic medical disease or malignancy^40^. Finally, 52 patients with NTM-PD (13 each with *M. avium*, *M. intracellulare*, *M. abscessus*, and *M. massiliense* infection) were selected and met the diagnostic criteria for NTM-PD^41^. As healthy controls, 43 hospital employees with no comorbidities were selected. Sera from the study subjects were separated by centrifugation and stored at −80 °C for subsequent analysis. Patient recruitment and informed consent were carried out in accordance with approved guidelines from the Institutional Review Board of Samsung Medical Center (IRB No. SMC-2008-09-016).

This study was performed in several phases to identify serum miRNAs that may be useful as markers of NTM-PD (Supplementary Fig. S3). Initially, in the discovery phase, screening was conducted by Illumina small RNA sequencing to select miRNAs with expression patterns that differed between patients with NTM-PD and control subjects. In this step, 15 serum samples were tested to obtain miRNA profiles, with the sera including three samples from patients with NTM for each of the four different pathogens, for a total of 12 patient samples, as well as three samples from control subjects. A panel of miRNAs showing differential expression in patients with NTM-PD and common to PD caused by all four pathogens was subjected to further testing by quantitative reverse-transcription (RT)-polymerase chain reaction (PCR) analysis using sera from additional patients and control subjects.

### RNA extraction

Total RNA was isolated from sera using a miRNeasy Mini Kit (Qiagen, Hilden, Germany) following the manufacturer’s protocol. Each sample was eluted in RNase-free water. RNA quantity and purity were measured using a NanoDrop™ ND-2000 (Thermo Fisher Scientific, Waltham, MA, USA), and RNA integrity was assessed using an Agilent Bioanalyzer 2100 (Agilent Technologies, Santa Clara, CA, USA). The RNA samples were stored at −80 °C until further analysis.

### Illumina high-throughput sequencing

Small RNA libraries were constructed using the NEXTFlex Small RNA Library Prep Kit (Illumina, San Diego, CA, USA) according to the manufacturer’s instructions. Briefly, an RNA sequencing library was prepared by cDNA amplification, end-repair, adenylylation of 3′ ends, adapter ligation, and amplification. The libraries were sequenced using the HiSeq 2500 system (Illumina) as 50-base pair single reads.

### Small RNA sequencing data analysis

The read data were assessed and pre-processed using FastQC (http://www.bioinformatics.babraham.ac.uk/projects/fastqc), and low-quality bases were trimmed from the 3′ end. Adapter sequences were trimmed and reads shorter than 17 nucleotides were discarded. Trimmed reads were mapped to the reference genome (GRCH38/hg19) using Bowtie^42^. The distribution of mapped reads was analyzed by crosschecking with miRBase (http://www.mirbase.org/), Ensembl annotations, and the Rfam database (http://rfam.xfam.org/). Finally, the miRDeep2 program^43^ (https://www.mdc-berlin.de/content/mirdeep2-documentation) was used to identify known miRNAs (based on miRNA data from miRBase v20) to predict novel miRNAs and summarize the read counts for each miRNA in every sample. Differentially expressed miRNAs were identified with a threshold fold-change greater than two and adjusted *p*-value < 0.05. *p*-values were calculated with the DESeq algorithm in the R package for experiments with biological replicates^44^.

### Quantification of miRNAs by qRT-PCR

Reverse transcription reactions were performed using a Taq-Man miRNA Reverse Transcription kit (Applied Biosystems, Foster City, CA, USA) and miRNA-specific stem-loop primers in accordance with the manufacturer’s instructions. Each reaction mixture for qRT-PCR contained 2.5 μL of 2 × TaqMan Universal PCR Master Mix without AmpErase UNG, 0.25 μL miRNA-specific primer/probe mix, and 2.25 μL diluted RT product (1:15) in a total volume of 5 μL. Reactions were carried out using the following thermal cycling parameters: 95 °C for 10 min, followed by 40 cycles of 95 °C for 15 s, and 60 °C for 1 min, followed by holding at 4 °C. Raw data were analyzed using SDS Relative Quantification Software version 2.2.3 (Applied Biosystems), generally using the automatic Ct setting for assigning baseline and threshold values for Ct determination. The expression level of each target miRNA was normalized to that of miR-16 (internal control).

### miRNA-gene network construction

TargetScan (http://genes.mit.edu/targetscan/index.html) was used to predict genes targeted by the obtained miRNAs. Target genes associated with NTM-PD were screened using the NCBI database (http://www.ncbi.nlm.nih.gov/pubmed/) and Cytoscape (Cytoscape Software, Version 2.8.2, Seattle, WA, USA). The miRNA gene network was constructed using Cytoscape to analyze miRNA-mRNA interactions.

### GO analysis and KEGG pathway analysis

GO enrichment was performed for analysis of the biological process, cellular component, and molecular function of target genes based on the GO database (http://www.geneontology.org/). KEGG pathway analysis was performed to identify the enriched pathways of target genes based on the KEGG database (http://www.genome.jp/kegg/). In addition, KEGG pathway annotation of target genes was found using data from the Database for Annotation, Visualization and Integrated Discovery (DAVID) (http://david.abcc.ncifcrf.gov/).

### Statistical analysis

Data are presented as the number (%) for categorical variables and median (interquartile range) for continuous variables. The data were statistically analyzed using SPSS software, version 17.0 (SPSS, Inc., Chicago, IL, USA) or the programs built into the software. Briefly, two-tailed unpaired *t*-tests were performed for one between-group comparison, and one-way analysis of variance was performed for multiple-group comparisons. Receiver operating characteristic (ROC) curve analysis was performed to evaluate the discriminative factor of the miRNAs. The AUC with 95% confidence interval (CI) were calculated to determine the specificity and sensitivity of discriminative markers of NTM-PD. ROC curve and logistic regression model were calculated by using the MedCalc Software (Version 19.1.13, Belgium). A default *p* < 0.05 was considered statistically significant.

## Supplementary information

Supplementary information

## Data Availability

All data generated or analysed during this study are included in this published article and its Supplementary Information Files.
