# Supplementary material for: miRNA Expression Profiles and Potential as Biomarkers in Nontuberculous Mycobacterial Pulmonary Disease
Source: Sci Rep. 2020 Feb 21;10:3178. doi: 10.1038/s41598-020-60132-0 (PMC7035291; doi:10.1038/s41598-020-60132-0)
Supplement: Supplementary file 1 — Supplementary information [file 41598_2020_60132_MOESM1_ESM.docx]

**miRNA Expression Profiles and Potential as Biomarkers in Nontuberculous Mycobacterial Pulmonary Disease**

Sun Ae Han, Byung Woo Jhun, Su-Young Kim, Seong Mi Moon, Bumhee Yang, O Jung Kwon, Charles L. Daley, Sung Jae Shin & Won-Jung Koh


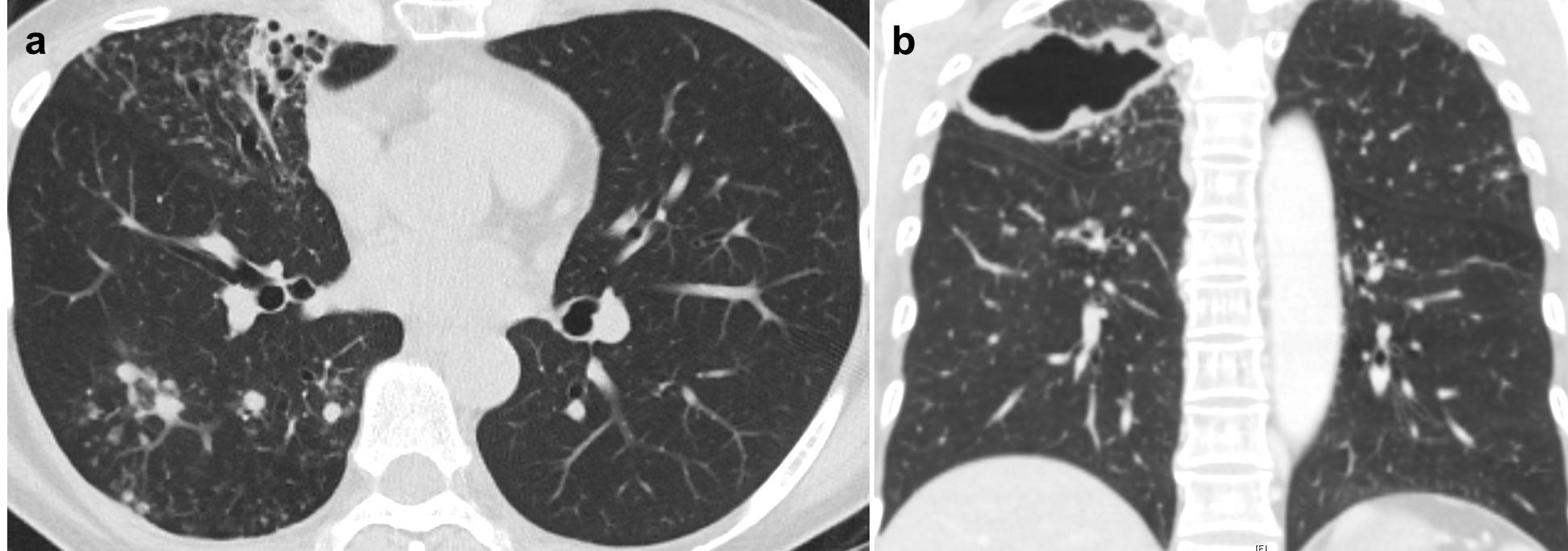


**Supplementary Figure S1.** The nodular bronchiectatic form of nontuberculous mycobacterial pulmonary disease in a 50 year old female patient (a). The fibrocavitary form of nontuberculous mycobacterial pulmonary disease in a 64 year old female patient (b).


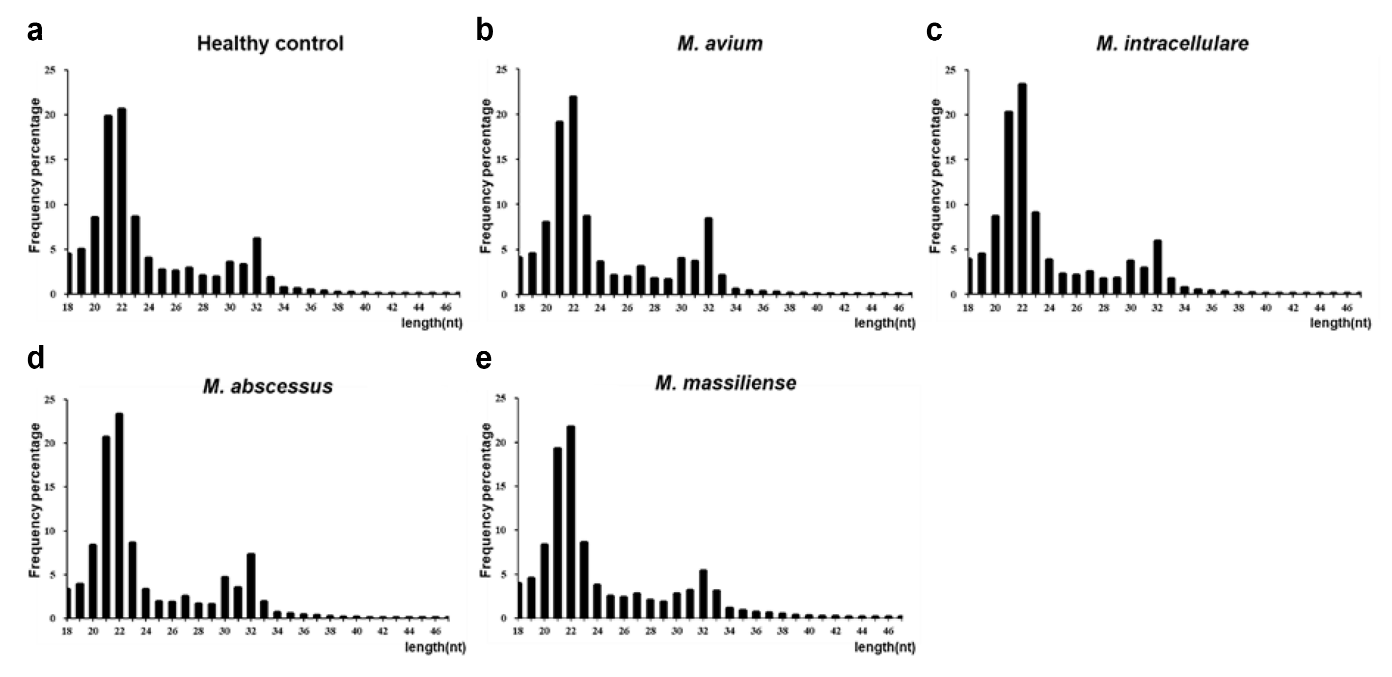


**Supplementary Figure S2.** Analysis of the length distribution of serum small RNAs. (**a**) healthy controls, (**b**) *Mycobacterium avium* patients, (**c**) *M. intracellulare* patients, (**d**) *M. abscessus* patients, (**e**) *M. massiliense* patients. The x-axis represents the length of clean reads, and the y-axis indicates the frequency percentage of different length reads.

**
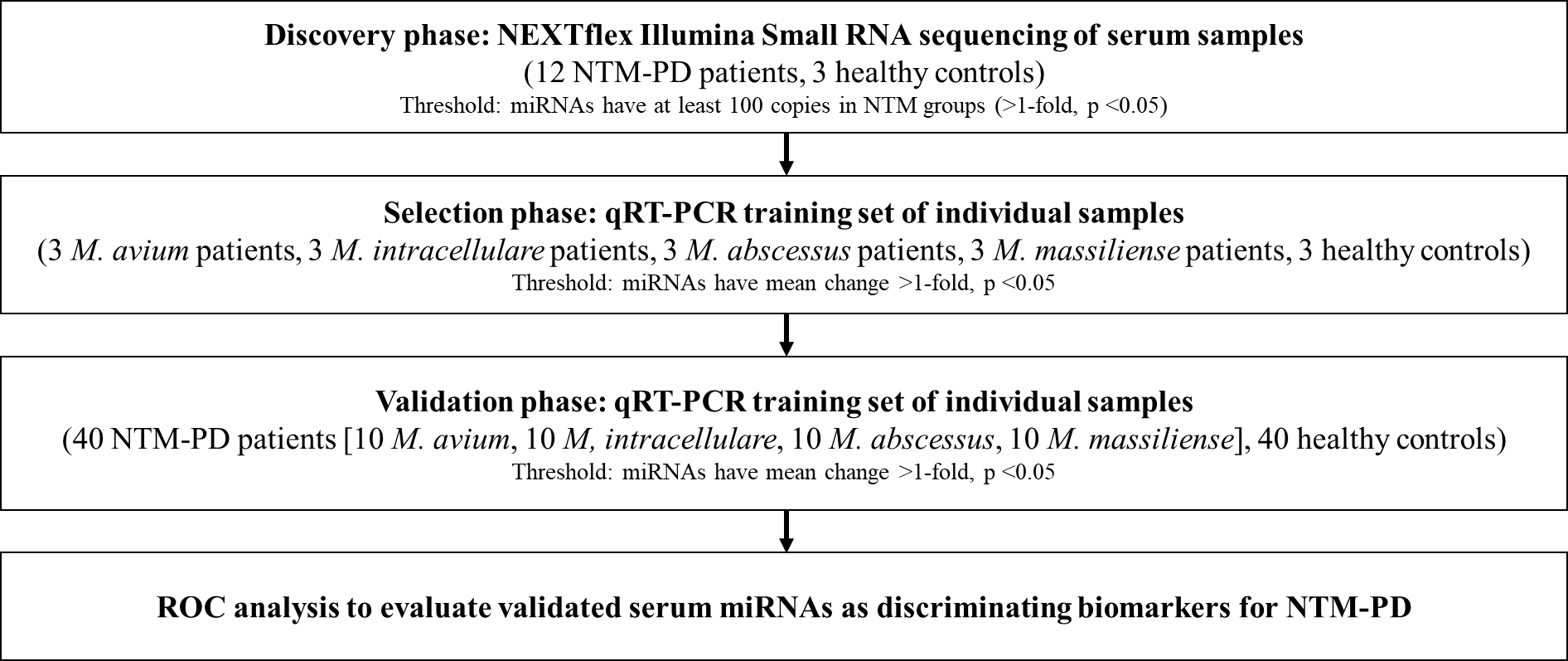
**

**Supplementary Figure S3.** A flow chart representing study design. miRNAs that were significantly differentially expressed between NTM-PD patients and healthy controls in the discovery set were included in the selection and validation sets. NTM-PD: nontuberculous mycobacterial pulmonary disease; qRT-PCR: quantitative reverse transcription polymerase chain reaction.

**Supplementary Table S1**. Differentially expressed miRNAs in patients with *Mycobacterium avium* pulmonary disease versus healthy controls. miRNAs with significantly altered expression in pooled serum samples of patients with *Mycobacterium avium* pulmonary disease compared to samples from healthy controls (*p* < 0.05; ordered by fold change) are shown.

| Down-regulated miRNAs | Fold change | Up-regulated miRNAs | Fold change |
| --- | --- | --- | --- |
| hsa-miR-1302 | 0.342 | hsa-miR-885-3p | 7.165 |
| hsa-miR-548x-5p | 0.225 | hsa-miR-4785 | 6.750 |
| hsa-miR-548g-5p | 0.222 | hsa-miR-1976 | 6.570 |
| hsa-miR-548aj-5p | 0.222 | hsa-miR-1228-5p | 6.563 |
| hsa-miR-548ar-5p | 0.222 | hsa-miR-99b-3p | 6.373 |
| hsa-miR-1179 | 0.128 | hsa-miR-3177-3p | 6.177 |
| hsa-miR-548h-5p | 0.119 | hsa-miR-6884-5p | 5.840 |
| hsa-miR-6503-5p | 0.116 | hsa-miR-211-5p | 5.793 |
| hsa-miR-411-5p | 0.101 | hsa-miR-323b-3p | 5.598 |
| hsa-miR-3614-3p | 0.098 | hsa-miR-5010-5p | 4.998 |
| hsa-miR-2115-5p | 0.094 | hsa-miR-654-5p | 4.910 |
|  |  | hsa-miR-939-5p | 4.823 |
|  |  | hsa-miR-1226-3p | 4.660 |
|  |  | hsa-miR-3679-5p | 4.407 |
|  |  | hsa-miR-4732-5p | 4.394 |
|  |  | hsa-miR-483-3p | 4.318 |
|  |  | hsa-miR-133a-3p | 4.044 |
|  |  | hsa-miR-2116-3p | 3.857 |
|  |  | hsa-miR-135a-5p | 3.793 |
|  |  | hsa-miR-376a-3p | 3.492 |
|  |  | hsa-miR-329-3p | 3.432 |
|  |  | hsa-miR-99b-5p | 3.269 |
|  |  | hsa-miR-125a-5p | 3.131 |
|  |  | hsa-miR-9-3p | 3.107 |
|  |  | hsa-miR-323a-3p | 3.085 |
|  |  | hsa-miR-885-5p | 3.002 |
|  |  | hsa-miR-584-5p | 2.977 |
|  |  | hsa-miR-4732-3p | 2.699 |
|  |  | hsa-miR-6803-3p | 2.621 |
|  |  | hsa-miR-1294 | 2.516 |
|  |  | hsa-miR-486-5p | 2.501 |
|  |  | hsa-miR-6511b-5p | 2.437 |
|  |  | hsa-miR-125b-5p | 2.359 |
|  |  | hsa-miR-625-3p | 2.274 |
|  |  | hsa-miR-4685-3p | 2.266 |
|  |  | hsa-miR-548a-3p | 2.210 |
|  |  | hsa-miR-92a-3p | 2.207 |
|  |  | hsa-miR-139-5p | 2.052 |
|  |  | hsa-miR-432-5p | 2.038 |
|  |  | hsa-miR-1285-3p | 1.978 |
|  |  | hsa-miR-10b-5p | 1.937 |
|  |  | hsa-miR-92b-3p | 1.847 |
|  |  | hsa-miR-181a-2-3p | 1.841 |
|  |  | hsa-miR-2110 | 1.834 |
|  |  | hsa-let-7d-3p | 1.809 |
|  |  | hsa-miR-122-5p | 1.718 |
|  |  | hsa-miR-877-5p | 1.692 |
|  |  | hsa-miR-10a-5p | 1.653 |
|  |  | hsa-miR-1306-5p | 1.631 |
|  |  | hsa-miR-382-5p | 1.598 |
|  |  | hsa-miR-28-3p | 1.592 |
|  |  | hsa-miR-483-5p | 1.579 |
|  |  | hsa-miR-501-3p | 1.533 |
|  |  | hsa-miR-409-3p | 1.527 |
|  |  | hsa-miR-320a | 1.476 |
|  |  | hsa-miR-30a-5p | 1.456 |
|  |  | hsa-miR-30d-5p | 1.413 |
|  |  | hsa-miR-423-5p | 1.398 |
|  |  | hsa-miR-484 | 1.394 |

**Supplementary Table S2.** Differentially expressed miRNAs in patients with *Mycobacterium intracellulare* pulmonary disease versus healthy controls. miRNAs with significantly altered expression in pooled serum samples of patients withSMC *Mycobacterium intracellulare* pulmonary disease compared to samples from healthy controls (p < 0.05; ordered by fold change) are shown.

| **Down-regulated miRNAs** | **Fold change** | **Up-regulated miRNAs** | **Fold change** |
| --- | --- | --- | --- |
| hsa-miR-548d-5p | 0.358 | hsa-miR-885-3p | 7.763 |
| hsa-miR-548ay-5p | 0.358 | hsa-miR-3679-5p | 5.910 |
| hsa-miR-1302 | 0.342 | hsa-miR-939-5p | 5.760 |
| hsa-miR-548x-5p | 0.225 | hsa-miR-642a-5p | 5.687 |
| hsa-miR-548g-5p | 0.225 | hsa-miR-6786-3p | 5.457 |
| hsa-miR-548aj-5p | 0.225 | hsa-miR-3177-3p | 5.357 |
| hsa-miR-548ar-5p | 0.225 | hsa-miR-548aq-3p | 4.311 |
| hsa-miR-548h-5p | 0.125 | hsa-miR-1275 | 4.297 |
| hsa-miR-2355-5p | 0.118 | hsa-miR-5010-5p | 4.232 |
| hsa-miR-6503-5p | 0.116 | hsa-miR-6885-5p | 4.163 |
|  |  | hsa-miR-133a-3p | 4.120 |
|  |  | hsa-miR-3605-3p | 4.075 |
|  |  | hsa-miR-9-3p | 3.741 |
|  |  | hsa-miR-3130-3p | 3.690 |
|  |  | hsa-miR-99b-5p | 3.189 |
|  |  | hsa-miR-4732-5p | 3.159 |
|  |  | hsa-miR-4732-3p | 3.159 |
|  |  | hsa-miR-125a-5p | 2.892 |
|  |  | hsa-miR-6859-5p | 2.853 |
|  |  | hsa-miR-584-5p | 2.813 |
|  |  | hsa-miR-885-5p | 2.789 |
|  |  | hsa-miR-625-3p | 2.527 |
|  |  | hsa-miR-486-5p | 2.476 |
|  |  | hsa-miR-28-3p | 2.303 |
|  |  | hsa-miR-139-5p | 2.171 |
|  |  | hsa-let-7d-3p | 2.131 |
|  |  | hsa-miR-2110 | 2.065 |
|  |  | hsa-miR-424-3p | 2.044 |
|  |  | hsa-miR-92a-3p | 2.017 |
|  |  | hsa-miR-484 | 1.986 |
|  |  | hsa-miR-365b-3p | 1.904 |
|  |  | hsa-miR-365a-3p | 1.904 |
|  |  | hsa-miR-181a-2-3p | 1.863 |
|  |  | hsa-miR-6511b-5p | 1.803 |
|  |  | hsa-miR-125b-5p | 1.788 |
|  |  | hsa-miR-193a-5p | 1.772 |
|  |  | hsa-miR-483-5p | 1.658 |
|  |  | hsa-miR-30d-5p | 1.646 |
|  |  | hsa-miR-4286 | 1.635 |
|  |  | hsa-miR-122-5p | 1.632 |
|  |  | hsa-miR-877-5p | 1.513 |
|  |  | hsa-miR-197-3p | 1.500 |
|  |  | hsa-miR-328-3p | 1.476 |
|  |  | hsa-miR-423-5p | 1.453 |
|  |  | hsa-miR-486-3p | 1.435 |
|  |  | hsa-miR-501-3p | 1.378 |

**Supplementary Table S3.** Differentially expressed miRNAs in patients with *Mycobacterium abscessus* pulmonary disease versus healthy controls. miRNAs with significantly altered expression in pooled serum samples of patients with *Mycobacterium abscessus* pulmonary disease compared to samples from healthy controls (p < 0.05; ordered by fold change) are shown.

| **Down-regulated miRNAs** | **Fold change** | **Up-regulated miRNAs** | **Fold change** |
| --- | --- | --- | --- |
| hsa-miR-548x-5p | 0.225 | hsa-miR-323b-3p | 19.510 |
| hsa-miR-548g-5p | 0.225 | hsa-miR-3679-5p | 13.520 |
| hsa-miR-548aj-5p | 0.225 | hsa-miR-654-5p | 11.320 |
| hsa-miR-548ar-5p | 0.225 | hsa-miR-3143 | 8.650 |
| hsa-miR-548h-5p | 0.119 | hsa-miR-7854-3p | 8.353 |
| hsa-miR-491-5p | 0.078 | hsa-miR-1304-5p | 7.907 |
|  |  | hsa-miR-8072 | 7.523 |
|  |  | hsa-miR-3177-3p | 6.823 |
|  |  | hsa-miR-1226-3p | 6.480 |
|  |  | hsa-miR-1908-5p | 6.224 |
|  |  | hsa-miR-135a-5p | 6.070 |
|  |  | hsa-miR-323a-3p | 6.070 |
|  |  | hsa-miR-5193 | 5.621 |
|  |  | hsa-miR-376a-3p | 5.236 |
|  |  | hsa-miR-6859-5p | 5.173 |
|  |  | hsa-miR-133a-3p | 4.610 |
|  |  | hsa-miR-329-3p | 3.824 |
|  |  | hsa-miR-584-5p | 3.723 |
|  |  | hsa-miR-3198 | 3.613 |
|  |  | hsa-miR-433-3p | 3.526 |
|  |  | hsa-miR-485-3p | 3.420 |
|  |  | hsa-miR-485-5p | 3.418 |
|  |  | hsa-miR-543 | 3.400 |
|  |  | hsa-miR-432-5p | 3.366 |
|  |  | hsa-miR-382-5p | 3.366 |
|  |  | hsa-miR-4732-5p | 3.143 |
|  |  | hsa-miR-99b-5p | 3.133 |
|  |  | hsa-miR-877-5p | 2.814 |
|  |  | hsa-miR-409-3p | 2.748 |
|  |  | hsa-miR-1285-3p | 2.587 |
|  |  | hsa-miR-4433b-3p | 2.562 |
|  |  | hsa-miR-625-3p | 2.527 |
|  |  | hsa-miR-28-3p | 2.491 |
|  |  | hsa-let-7e-5p | 2.339 |
|  |  | hsa-miR-328-3p | 2.278 |
|  |  | hsa-let-7d-3p | 2.168 |
|  |  | hsa-miR-2110 | 2.031 |
|  |  | hsa-miR-484 | 2.007 |
|  |  | hsa-miR-125a-5p | 1.982 |
|  |  | hsa-miR-423-5p | 1.897 |

**Supplementary Table S4.** Differentially expressed miRNAs in patients with *Mycobacterium massiliense* pulmonary disease versus healthy controls. miRNAs with significantly altered expression in pooled serum samples of patients with *Mycobacterium massiliense* pulmonary disease compared to samples from healthy controls (p < 0.05; ordered by fold change) are shown.

| **Down-regulated miRNAs** | **Fold change** | **Up-regulated miRNAs** | **Fold change** |
| --- | --- | --- | --- |
| hsa-miR-1302 | 0.342 | hsa-miR-3679-5p | 17.550 |
| hsa-miR-548x-5p | 0.225 | hsa-miR-374b-3p | 10.630 |
| hsa-miR-548g-5p | 0.225 | hsa-miR-570-3p | 7.710 |
| hsa-miR-548aj-5p | 0.225 | hsa-miR-654-5p | 7.633 |
| hsa-miR-548ar-5p | 0.225 | hsa-miR-323b-3p | 7.567 |
| hsa-miR-1273c | 0.134 | hsa-miR-7854-3p | 7.357 |
| hsa-miR-1179 | 0.128 | hsa-miR-4732-5p | 6.271 |
| hsa-miR-548h-5p | 0.125 | hsa-let-7c-3p | 6.250 |
| hsa-miR-3614-3p | 0.098 | hsa-miR-3177-3p | 6.117 |
|  |  | hsa-miR-369-5p | 6.100 |
|  |  | hsa-miR-8072 | 5.423 |
|  |  | hsa-miR-625-3p | 3.177 |
|  |  | hsa-miR-1285-3p | 2.733 |
|  |  | hsa-miR-584-5p | 2.315 |
|  |  | hsa-miR-550a-3-5p | 2.274 |
|  |  | hsa-miR-99b-5p | 2.141 |
|  |  | hsa-miR-125a-5p | 2.074 |
|  |  | hsa-let-7e-5p | 2.054 |
|  |  | hsa-miR-550a-5p | 1.962 |
|  |  | hsa-miR-423-5p | 1.897 |
|  |  | hsa-miR-320a | 1.813 |
|  |  | hsa-miR-486-5p | 1.781 |
|  |  | hsa-miR-484 | 1.707 |
